# Ezrin promotes invasion and metastasis of pancreatic cancer cells

**DOI:** 10.1186/1479-5876-8-61

**Published:** 2010-06-23

**Authors:** Yunxiao Meng, Zhaohui Lu, Shuangni Yu, Qiang Zhang, Yihui Ma, Jie Chen

**Affiliations:** 1 Department of Pathology, Peking Union Medical College Hospital, Chinese Academy of Medical Sciences and Peking Union Medical College, Tsinghua University, 1 Shuai Fu Yuan Hu Tong, Beijing, China

## Abstract

**Background:**

Pancreatic cancer has a high mortality rate because it is usually diagnosed when metastasis have already occurred (microscopic and gross disease). Ezrin plays important roles in cell motility, invasion and tumor progression, and it is especially crucial for metastasis. However, its function in pancreatic cancer remains elusive.

**Methods and Results:**

We found that ezrin overexpression promoted cell protrusion, microvillus formation, anchorage-independent growth, motility and invasion in a pancreatic cancer cell line, MiaPaCa-2, whereas ezrin silencing resulted in the opposite effects. Ezrin overexpression also increased the number of metastatic foci (6/8 *vs*. 1/8) in a spontaneous metastasis nude mouse model. Furthermore, ezrin overexpression activated Erk1/2 in MiaPaCa-2 cells, which might be partially related to the alteration of cell morphology and invasion. Immunohistochemical analysis showed that ezrin was overexpressed in pancreatic ductal adenocarcinoma (PDAC) (91.4%) and precancerous lesions, i.e. the tubular complexes in chronic pancreatitis (CP) and pancreatic intraepithelial neoplasm (PanIN) (85.7% and 97.1%, respectively), compared to normal pancreatic tissues (0%). Ezrin was also expressed in intercalated ducts adjacent to the adenocarcinoma, which has been considered to be the origin of ducts and acini, as well as the starting point of pancreatic ductal carcinoma development.

****Conclusions**:**

We propose that ezrin might play functional roles in modulating morphology, growth, motility and invasion of pancreatic cancer cells, and that the Erk1/2 pathway may be involved in these roles. Moreover, ezrin may participate in the early events of PDAC development and may promote its progression to the advanced stage.

## Background

Ezrin, encoded by the *Vil2 *gene, is a member of the ERM family; it provides a functional link between the plasma membrane and the cortical actin cytoskeleton of the cell. Ezrin plays important roles in cell motility, morphogenesis, adhesion, survival and apoptosis [1-6]. It also participates in crucial signal transduction pathways [7]. Ezrin binds to cell surface glycoproteins, such as CD43, CD44, ICAM-1 and ICAM-2, through interacting with their amino (N)-terminal domains. Ezrin also binds to filamentous actin through its carboxyl (C)-terminal domains [8]. Ezrin has been linked to molecules that control the phosphatidylinositol-3-kinase, AKT, Erk1/2 MAPK and Rho pathways, which are functionally involved in signaling events regulating cell survival, proliferation and migration. Phosphorylation of ezrin induces its translocation from the cytoplasm to the plasma membranes of microvillus and confers the ability of binding to plasma membrane and actin filaments [9-12].

Ezrin is expressed in a variety of normal and neoplastic cells, including many types of epithelial, lymphoid and glial cells [5,13,14]. In melanoma cells, ezrin has been shown to be localized in phagocytic vacuoles, suggesting that its association with the actin cytoskeleton is crucial for the phagocytic activity [15]. Phagocytic behavior is usually considered to be an indicator of high-grade malignancy in melanomas. In addition, immunohistochemical analysis has demonstrated a significant correlation between increased ezrin immunoreactivity and a high histological grade in astrocytoma [16]. In a complementary DNA (cDNA) microarray analysis of highly and poorly metastatic rhabdomyosarcomas, ezrin was indicated to be a key regulator of metastasis [17]. Ezrin overexpression has also been considered as an independent predictor of adverse outcome of gastrointestinal stromal tumors [18]. These results indicated that ezrin expression level is closely associated with malignant progression of cancer.

Consistent with these reports, suppression of ezrin protein expression and disruption of its function significantly reduced lung metastasis in a mouse osteosarcoma model [19]. Furthermore, high-level ezrin expression in canine osteosarcomas has been associated with early development of metastasis [20]. Ezrin silencing by small hairpin RNA could reverse the metastatic behavior of human breast cancer cells [21]. Taken together, the observed effects of ezrin overexpression and silencing on the cell malignant transformation indicate a role for ezrin in regulating tumor metastasis and progression [22].

In pancreatic carcinomas, a high-level ezrin expression is associated with high metastatic potential; membrane translocation of ezrin might play a role in the progression from borderline tumor to malignant transformation. Patients with pancreatic ductal adenocarcinoma (PDAC) with membranous ezrin expression exhibited poorer prognosis compared to those without membranous ezrin expression, and ERM protein was more likely to be present in poorly differentiated cancers [23-26]. A recent study showed that overexpression of pEzrin (Tyr353) in pancreatic cancers was associated with positive lymph node metastasis, less differentiation, pAkt overexpression and shorter survival times [27]. Ezrin can interact with cortactin to form podosomal rosettes in pancreatic cancer cells, thereby playing a role in pancreatic cancer invasion [28]. However, the mechanisms of ezrin-mediated tumor development still require further elucidation. In this study, we investigated the effect of ezrin on the motility and invasion ability of the pancreatic cancer cell line MiaPaCa-2, as well as the expression of ezrin in pancreatic duct adenocarcinoma, chronic pancreatitis and normal pancreatic tissues.

## Materials and methods

### Antibodies and plasmids

Rabbit polyclonal anti-ezrin antibody was purchased from Upstate technology (Lake Placid, NY). Rabbit polyclonal anti-phosphorylated Ezrin (Tyr353), mouse monoclonal anti-AKT, anti-phospho-AKT (Ser473), anti-p44/42 MAPK (Erk1/2) and anti-phospho-p44/42 MAPK (Erk1/2) (Thr202/Tyr204) antibodies were purchased from Cell Signaling Technology (Beverly, MA, USA). The mouse monoclonal antibody VSV-G (P5D4) was purchased from Roche Applied Science (Indianapolis, USA). The mouse monoclonal antibody GAPDH was purchased from Santa Cruz Biotechnology (Santa Cruz, CA). The secondary antibodies, including the rhodamine-conjugated goat anti-mouse, FITC-conjugated goat anti-mouse, horseradish peroxidase-conjugated anti-mouse and anti-rabbit antibodies, were purchased from ZhongShan Biotechnology (Beijing, China). The pcb6 vector that contains the cDNA encoding VSV-G-tagged ezrin was kindly provided by Dr. Monique Arpin [14].

### Plasmid-based silencing of ezrin expression

The mammalian expression vector, pSilencer 2.1-U6 (Ambion, Austin, Texas, USA) was used for expressing of siRNA in MiaPaCa-2 cells. Briefly, two primer pairs were synthesized, with the first pair encoding the nucleotides, GGGCCAAGTTCTACCCTGAAG (376-396, No. 1) followed by a 9 base "loop", TTCAAGAGA and an inverted repeat and the second pair encoding the nucleotides, GGCTTTCCTTGGAGTGAAA (849-867, No. 2) followed by the loop and the inverted repeat. A nonspecific 21-nucleotide siRNA scrambled to the first pair, GACCGAGTCCGAAGTCAGCT (No. 3) was used as a control. The primer pairs were annealed and inserted into the BamH I and Hind III sites of pSilencer 2.1-U6 and transformed into JM109 competent cells (Promega, Madison, WI, USA). Positive clones were identified and verified by restriction enzyme analysis and sequence analysis.

### Cell culture and cell transfection

The pancreatic adenocarcinoma cell line MiaPaCa-2 (American Type Culture Collection, Manassas, Virginia, USA) was grown in DMEM (GIBCO, Grand Island, New Yolk, USA) supplemented with 10% fetal calf serum (FCS) and 1% L-glutamine (Invitrogen, Karlsruhe, Germany) and maintained at 37°C in 5% CO_2_. All transfections reactions were performed using Lipofectamine 2000 (Invitrogen; Carlsbad, CA) in accordance with the manufacturer's instructions. Stable transfectants were selected with 800 μg/mL G418 (Sigma-Aldrich, St. Louis, MO, USA), and individual clones were isolated.

### Scanning electron microscopy

Cells were cultured on coverslips and harvested after 24 hours. Cells were then washed with phosphate buffered saline (PBS) and fixed with 2.5% glutaraldehyde at 4°C for 12 hours. After thoroughly washing with PBS, the fixed cells were dehydrated through an ethanol series and dried at room temperature. The samples were coated with a thin film of silver and examined under a scanning electron microscope (JEOL/JSM-6000F, JEOL Ltd., Tokyo, Japan).

### Western blotting

Cell lysates (30 μg protein) resolved on 10% SDS-PAGE were transferred to a polyvinylidene difluoride membrane (Millipore, Bedford, MA). For immunoblotting, we used antibodies against ezrin, VSV-G, phospho-ezrin(Tyr353), phospho-p44/42 MAPK(Erk1/2) (Thr202/Tyr204), p44/42 MAPK (Erk1/2), phospho-AKT (Ser473), AKT and GAPDH. The immunoreactive proteins were visualized using the ECL western blotting system (Amersham International, little Chalfont, UK), and densitometric analysis was performed using the Image Pro-Plus Software.

### Indirect immunofluorescence

Cells were plated on glass coverslips for 24 hours, fixed with 3.7% paraformaldehyde for 20 minutes and then permeabilized with PBS containing 0.05% Triton X-100 for 10 minutes. The cells were then blocked with 1% BSA in PBS for 1 hour, followed by adding of primary antibodies diluted in blocking buffer at 4°C overnight at the following concentrations: anti-ezrin (serum was diluted 1:150) and anti-VSV-G (serum was diluted 1:75). Subsequently, the cells were washed with PBS and then incubated for 1 hour in either the goat-anti-mouse IgG TRITC-conjugated antibodies or the goat-anti-rabbit IgG FITC-conjugated antibody, both of which were diluted in the blocking buffer (1:60). Afterwards, 4',6-diamidino-2-phenylindole (DAPI) was used for nuclear counter-staining. Finally, the cells were mounted in the fluorescent mounting medium (Applygen Technologies Inc., Beijing, China) and viewed with under a fluorescence microscope (BH2-RFCA; Olympus Optical Co., Ltd, Tokyo, Japan).

### Cell growth assay and flow cytometry analysis

*In vitro *cell growth was assessed using the Dojindo Cell Counting Kit-8 (Dojindo Laboratory, Kumamoto, Japan) according to the supplier's recommendations. Clones were plated in tissue culture plates at a density of 1 × 10^3 ^cells in 0.1 mL of culture medium per well and grown in DMEM with 10% FCS in 5% CO_2 _at 37°C. The number of cells per well was quantified by daily measurement of the absorbance at 450 nm for 7 days after plating. All experiments were performed in triplicate on three separate occasions. Replicate growth curves were plotted for each of the clones and compared to control cells grown under identical culture conditions. To determine the cell cycle distribution, 5 × 10^5 ^cells were plated in 60-mm dishes and cultured for periods of up to 2 days. The cells were then collected by trypsinization, fixed with 70% ethanol, washed with PBS, resuspended in 1 mL of 0.01 M PBS with RNase and 50 μg/mL propidium iodide, incubated for 20 minutes in the dark at room temperature and analyzed by flow cytometry using a FACS Calibur (Becton Dickinson, Bedford, MA).

### Colony formation assay

An equal amount of 1% Noble agar solution pre-warmed to 40°C was added to DMEM containing 20% FCS pre-warmed to 37°C to make a 0.5% agar solution. After rapid mixing by inversion, the resultant solution was added to 24-well plates (0.5 mL/well). After reaching 70 to 80% confluence, the cells were trypsinized, washed with D-Hanks three times and diluted in Noble agar solution (0.35% Noble agar in DMEM with 10% FCS) at 37°C. The cell suspensions were then added into 24-well plate with a 0.5% agar layer (200 cells in 0.5 mL) (three wells per condition). The plates were incubated at 37°C with 5% CO_2 _for three weeks. The colony formation ability under each condition was assessed using untreated cells as control.

### Transfilter migration and invasion assays

Transfilter assays were performed with 8.0-μm pore inserts in 24-well BioCoat Chambers (Becton Dickinson) using 5 × 10^4 ^cells in serum-free DMEM. The DMEM medium with 10% FCS was placed in the lower chambers as a chemoattractant. For invasion assays, Matrigel-coated transwell chambers were used. For migration and invasion assays, the cells were removed from the upper surface of the filter by scraping with a cotton swab after 12 and 24 hours in culture respectively. Migrated cells and invasive cells were fixed and stained with the crystal violet reagent. Mean values of the data obtained from three separate chambers were presented.

### Tumor transplantation and spontaneous/experimental metastasis

Female BALB/c nude mice (body weight, 15 to 17 g) were bred under specified pathogen-free conditions (26°C, 70% relative humidity and a 12-h light/12-h dark cycle) in a germ-free environment with free access to food and water. To examine the effects of ezrin on tumor cell proliferation and metastasis *in vivo*, Mia ez22-B, Mia pcb6, Mia ezsi-scram and Mia ezsi-E (5 × 10^6 ^cells/100 μL normal sodium/mouse) were used. For spontaneous metastasis, the cells were injected into the inferior of pancreas capsule of the nude mice, whereas for experimental metastasis, the cells were injected into the tail vein of the nude mice. The mice were monitored every 2 to 3 days and sacrificed 10 weeks after injection. Tumors were excised, and metastasis in the lung, viscera, liver, draining lymph nodes and other organs were assessed. These tumors were embedded into paraffin. Histological analysis of the tissue sections stained with hematoxylin and eosin were performed to confirm the presence of metastasis in the various organs. Based on the gross and histological analyses, animals were assessed as positive or negative with respect to metastasis. Animal handling and experimental procedures were approved by the Peking Union Medical College Hospital animal experiments committee. It was also in accordance with the recommendations by the regional and country animal ethics committee.

### Patients, specimens and immunohistochemistry

This study was approved by the Institutional Review Board of Peking Union Medical College Hospital, Chinese Academy of Medical Sciences (CAMS) and Peking Union Medical College (PUMC). Surgically resected specimens from 70 patients (age range, 29 to 78 years) with PDAC were examined. This patient population represented a randomly selected subgroup from a clinical series including all patients who underwent surgical resection between June 1998 and December 2005 in the Department of Surgery at Peking Union Medical College Hospital. The diagnosis of PDAC, histological grading and pathologic staging were re-evaluated and/or confirmed by two independent pathologists. PanIN lesions (n = 34) and CP (n = 28) were assessed and graded in the pancreatic tissues adjacent to the tumor in hematoxylin and eosin-stained slides.

Immunostaining for ezrin was performed using the primary rabbit polyclonal antibody against human ezrin (diluted 1:150) at 4°C overnight after antigen retrieval in 10 mM sodium citrate buffer (pH 6.0) for 15 minutes at 95°C, followed by incubation with an HRP-labeled anti-rabbit antibody for 1 hour. Immunostaining and clinicopathologic features were evaluated microscopically by two pathologists. Ezrin-specific immunoreactivity was scored by estimating the percentage of labeled tumor cells as follows: score 0, < 25% positive cancer cells; score +, 25-50% positive cancer cells; score ++, 50-75% positive cancer cells; and score +++, > 75% positive cancer cells. Specimens were considered positive for ezrin expression when the scores were + to +++ and were considered negative for ezrin expression when the score was 0. Pictures were collected using the MicroView MVC2000 image apparatus and software.

### Statistical analysis

Each experiment was performed three to four times. All of the data were expressed as mean ± SD. Statistical analysis was performed using the Microsoft Excel software package. Comparisons between groups were conducted using Welch's *t *test. Correlation of ezrin immunoreactivity with clinicopathologic parameters were analyzed by Fisher's exact test. Differences were considered statistically significant at *P *< 0.05.

## Results

### Establishment of ezrin overexpression monoclones and silencing of the ezrin gene in MiaPaCa-2 cells

**Figure 1:**
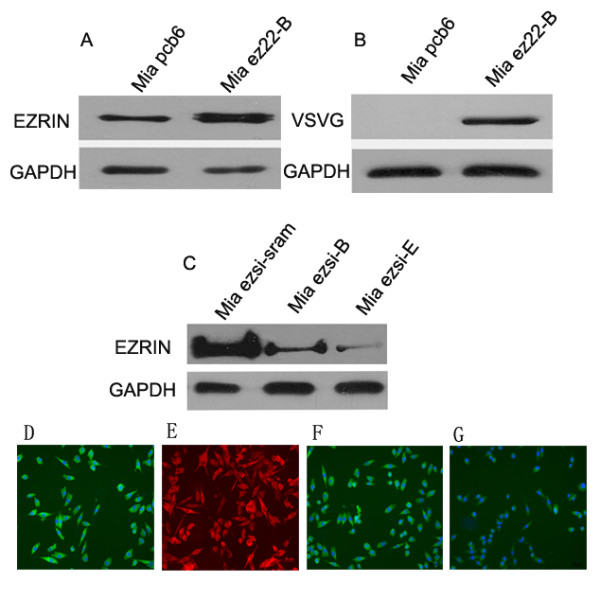
**Stable overexpression and silencing of ezrin in MiaPaCa-2 cells**. (A) Western blot showed the ezrin protein was overexpressed in the Mia ez22-B cells compared to the Mia pcb6 cells using an ezrin antibody. The relative ezrin protein level was quantified by densitometry analysis. The ezrin protein was efficiently increased by 3.8 folds in the Mia ez22-B cells. (B) Ectopic expression of ezrin in the Mia ez22-B cells was detected using a VSV-G antibody (VSV-G tag in the pcb6-ezrin vector). (C) The expression level of ezrin protein was dramatically decreased by 70.5% and 90.1% in the Mia ezsi-B and the Mia ezsi-E cells, respectively, compared to that in the Mia ezsi-scram cells. GAPDH was used as a loading control. (D) The Mia pcb6 cells were stained with the ezrin antibody and a FITC-conjugated second antibody to detect the ezrin protein expression. (E) The vector tag VSV-G antibody and a Rhodamine-conjugated second antibody were used to detect the exogenous ezrin protein expression. (F, G) The Mia ezsi-scram and the Mia ezsi-E cells were stained with the ezrin antibody and the FITC-conjugated second antibody to detect the ezrin protein expression.

To study the function of the *Vil2 *gene in MiaPaCa-2 cells, the pcb6-ezrin-VSV-G vector was adopted to stably overexpress the ezrin protein, and the pcb6 vector was used as a control. For ezrin silencing, the three ezrin siRNAs, described in the Materials and methods, were synthesized and transfected into MiaPaCa-2 cells. Western blot analysis showed the No. 2 siRNA inhibited ezrin more efficiently (data not shown). Thus, the No. 2 and No. 3 siRNA sequences were cloned into the pSilencer 2.1 U6 vector. G418-screened MiaPaCa-2 cells were used for analysis, and the stable cell clones Mia ez22-B, Mia pcb6, Mia ezsi-B, Mia ezsi-E and Mia ezsi-scram were selected. Western blot analysis showed that ezrin protein expression was efficiently increased by 3.8 folds in the Mia ez22-B cells compared to the Mia pcb6 cells (Figure 1A, B). It was also shown that ezrin protein expression was efficiently decreased by 70.5% and 90.1% in the Mia ezsi-B and Mia ezsi-E cells, respectively, compared to that in the Mia ezsi-scram cells (Figure 1C). In addition, immunoflurescence staining using the VSV-G-tagged ezrin antibody further confirmed its stable overexpression in the MiaPaCa-2 cells (Figure 1D, E), which also showed that ezrin protein expression was dramatically decreased in the Mia ezsi-E cells (Figure 1G) compared to that in the Mia ezsi-scam cells (Figure 1F).

### Ezrin overexpression enhancing the formation of cell protrusions and cell microvilli

**Figure 2:**
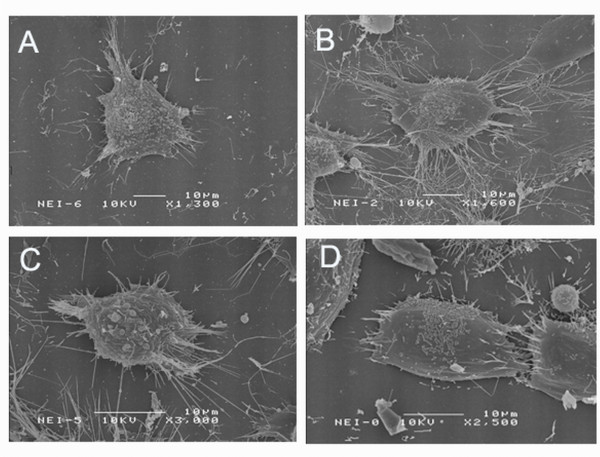
**Scanning electron microscopy showed increased formation of membrane protrusions and microvilli in the Mia ez22-B cells (B) compared to that in the Mia pcb6 cells (A)**. A sharp decrease of the membrane protrusions and smooth edge in the Mia ezsi-E cells (D) compared to those in the Mia ezsi-scram cells (C).

To explore whether ezrin is involved in cytoskeleton modulation, we studied the morphological changes of the stable transfectants by scanning electron microscopy (SEM). Compared to those in the Mia pcb6 cells, there was a sharp increase in the numbers of membrane protrusions and more elongated membrane projections in the Mia ez22-B cells (Figure 2B). The Mia pcb6 cells exhibited a smooth edge and fewer projections (Figure 2A). In contrast, compared to those in the Mia ezsi-scram cells, a dramatic decrease in the numbers of membrane protrusions and smooth edges were observed in the Mia ezsi-E cells (Figure 2D), and the Mia ezsi-scram cells showed more projections and more elongated membrane projections (Figure 2C). The morphologic changes suggest possible alteration of tumor cell behavior.

### Ezrin altering anchorage-independent growth ability without affecting cell proliferation or cell cycle distribution in vitro

**Figure 3:**
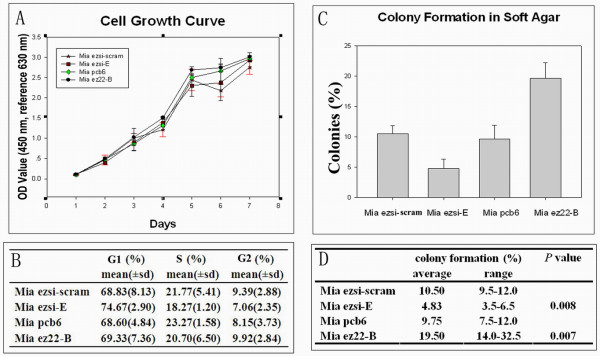
**Effects of ezrin on MiaPaCa-2 cell growth and anchorage-independent growth**. (A) The cell growth curves of the Mia ezsi-scram, Mia ezsi-E, Mia pcb6 and Mia ez22-B cells were assayed on days 1-7. (B) Flow cytometry assay showing the percentage of different cell cycle phases in the four cell clones. (C) Anchorage-independent growth assay of ezrin-overexpressing and ezrin-silencing cells. The cell growth ability in soft agar of the four cell clones was examined for three weeks. Columns, mean; bars, SD. (D) Statistical analysis of colony formation in the four cell clones. There was a significant difference of the colony formation ability between the Mia ez22-B and the Mia pcb6 cells, as well as between the Mia ezsi-scram and the Mia ezsi-E cells, respectively, shown by *x*^2^-test. The results are expressed as the mean ± SD of three independent experiments.

A series of experiments were conducted to determine the effect of different ezrin protein levels on the proliferation of MiaPaCa-2 cells *in vitro*. The effect of the ezrin protein on cell growth rate was examined by the CCK-8 assay. The change in the ezrin protein level had no significant effect on the cell growth rate *in vitro *(Figure 3A). The flow cytometry assay further showed that changes in the ezrin protein level did not affect the cell cycle distribution (Figure 3B). To further characterize the effect of ezrin on anchorage-independent growth ability, the colony forming assay was performed. Ezrin overexpression facilitated the anchorage-independent growth ability of the Mia ez22-B cells when compared to that of the Mia pcb6 cells, and ezrin silencing decreased the anchorage-independent growth ability in the Mia ezsi-E cells compared to that of the Mia ezsi-scram cells (Figure 3C). Statistical analysis showed that the anchorage-independent growth ability of the tumor cells in soft agar was increased by 103.1% in the Mia ez22-B cells compared to that in the Mia pcb6 cells, and it was decreased by 54.3% in the Mia ezsi-E cells compared to that in the Mia ezsi-scram cells (Figure 3D). These results indicated that ezrin could enhance the anchorage-independent growth ability of MiaPaCa-2 cells.

### Ezrin increasing the cell motility and invasion ability of MiaPaCa-2 cells

**Figure 4:**
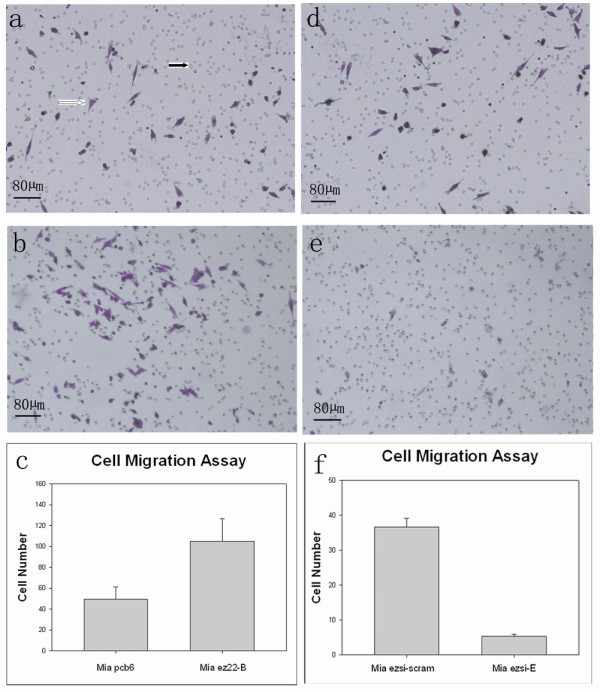
**Effects of ezrin on cell motility *in vitro***. BioCoat Chambers were used to detect cell migration and representative fields were photographed. Black-arrows indicate the 8-µm membrane pores, and hollow-arrows indicate cells that had migrated through the membrane, which were stained with Crystal Violet (a). Cell migration of the Mia ez22 (b), Mia pcb6 (a), Mia ezsi-E (e) and Mia ezsi-cram (d) cells after 12 hours were shown. The cells migrating to the lower chambers were analyzed. For quantification, the cells were counted in 10 random fields under a light microscope (×400). Compared to the Mia pcb6 cells, the Mia ez22-B cells showed a significant increase in migration by x^2^-test (c). The decrease in the numbers of migrated cells in the Mia ezsi-E cells compared to those of the Mia ezsi-scram cells was statistically significant, shown by the x^2^-test (f). Columns: mean; bars: SD.

Cell motility ability was examined by determining of the migration rate through a polyethylene filter in the absence of Matrigel. The migration rate of the Mia ez22-B cells (Figure 4b) was greatly increased compared to that of the Mia pcb6 cells (Figure 4a). The average cell number of the Mia ez22-B cells migrating to the lower chamber was 105 ± 5.06 per high-power field (0.312 mm^2^/HPF), compared to 40.4 ± 2.86/HPF of the Mia pcb6 cells. The quantitative analysis showed that cell migration to the lower chamber was increased by 1.59 folds in the Mia ez22-B cells compared to that in the Mia pcb6 cells (*P *< 0.01) (Figure 4c). Compared to that of the Mia ezsi-scram cells (Figure 4d), the migration rate of the Mia ezsi-E cells (Figure 4e) was greatly decreased. The average cell number of the Mia ezsi-E migrating to the lower chamber was 5.39 ± 0.32/HPF, compared to 36.7 ± 1.453/HPF of the Mia ezsi-scram cells. The quantitative analysis showed that cell migration to the lower chamber were decreased by 58.3% in the Mia ezsi-E cells compared to that in the Mia ezsi-scram cells (*P *= 0.00003) (Figure 4f).

**Figure 5:**
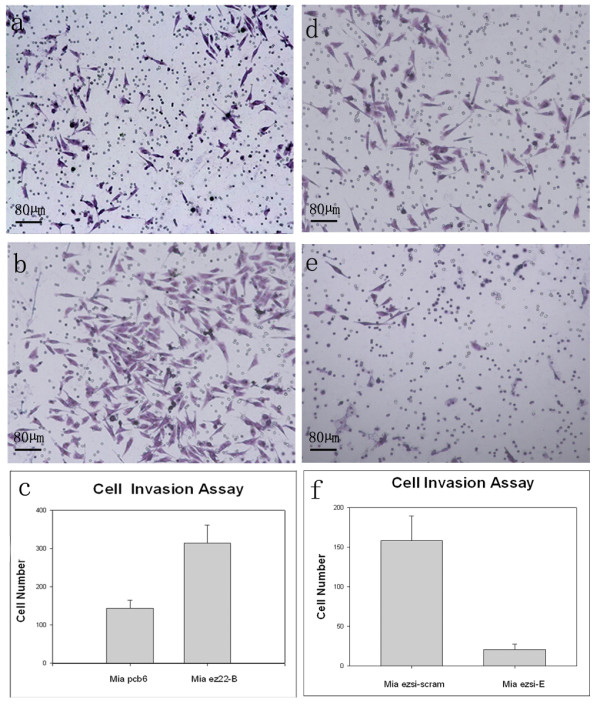
**Effects of ezrin on cell invasion* in vitro***. Matrigel-coated transwell chambers were used to detect cell invasion and representative fields were photographed. Cell invasion of the Mia ez22 (b), Mia pcb6 (a), Mia ezsi-E (e) and Mia ezsi-cram (d) cells after 24 hours were shown. The cells invading to the lower chambers were analyzed. Compared to the Mia pcb6 cells, the Mia ez22-B cells showed a significant increase in invasion by x^2^-test (c). The decrease in the numbers of invasive cells in the Mia ezsi-E cells compared to those of the Mia ezsi-scram cells was statistically significant, shown by the x^2^-test (f). Columns: mean; bars: SD.

We next examined whether ezrin can affect the invasion activity of pancreatic cancer cells by the Matrigel invasion assay. Cell invasive activity was also dramatically enhanced in the Mia ez22-B cells (Figure 5b) compared to that in the Mia Pcb6 cells (Figure 5a). The average cell number invading to the lower chamber for 24 hours was 314 ± 46.93/HPF in the Mia ez22-B cells, compared to 144 ± 20.42/HPF in the Mia pcb6 cells. The quantitative analysis demonstrated that the number of the Mia ez22-B cells invading to the lower chamber was increased by 1.18 folds compared to that of the Mia pcb6 cells (*P *= 0.0045) (Figure 5c). In addition, cell invasive activity was also dramatically decreased in the Mia ezsi-E cells (Figure 5e) compared to that in the Mia ezsi-scram cells (Figure 5d), which was 20.6 ± 4.06/HPF and 158 ± 17.85/HPF, respectively. The quantitative analysis showed that the number of Mia ezsi-E cells invading to the lower chamber was decreased by 87.0% compared to that of the Mia ezsi-scram cells (*P *= 0.0017) (Figure 5f). Both the increase and decrease of cell motility and invasion might result from morphological alterations of the MiaPaCa-2 cells, such as increased protrusions and microvilli.

### Ezrin overexpression inducing Erk1/2 activation

**Figure 6:**
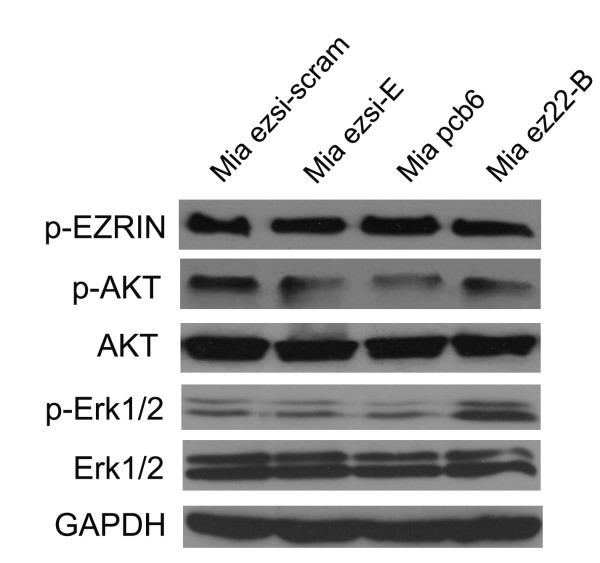
**Ezrin overexpression increasing the level of phosphorylated Erk1/2 in MiaPaCa-2 cells.** The levels of phosphorylated-ezrin, total AKT, phosphorylated-AKT, total Erk1/2 and phosphorylated Erk1/2 were determined by western blot in the Mia ezsi-scram, Mia ezsi-E, Mia pcb6 and Mia ez22-B cells. GAPDH was used as a loading control.

The results described above indicate that ezrin is involved in the motility and invasion of MiaPaCa-2 cells. Erk1/2 signaling has been shown to disrupt actin stress fibers, which in turn increases cell motility by changing actin dynamics and decreasing of cell adhesion [29]. The PI3-kinase pathway has also been shown to be responsible for RAC-dependent membrane ruffling downstream of the Ras signaling pathway [30]. It has been recently reported that phosphorylation of ezrin is required for metastatic behavior of tumor cells [31]. Our results showed that ezrin overexpression increased the level of phosphorylated-Erk1/2 protein without altering the level of total Erk1/2 in MiaPaCa-2 cells. However, there was no obvious alteration in the level of phosphorylated-Erk1/2 protein in the Mia ezsi-E cells. Those results suggest that the Erk1/2 pathway might participate in the ezrin-mediated cell growth, motility and invasion. Moreover, there were no obvious changes in the protein levels of Akt, phosphorylated-Akt and phosphorylated-ezrin (Tyr353) in both the ezrin silencing and the ezrin overexpression clones of MiaPaCa-2 cells (Figure 6).

### Ezrin overexpression promoting metastasis of MiaPaCa-2 cells in vivo

Tumorigenicity and metastasis of the Mia ez22-B, Mia pcb6, Mia ezsi-E and Mia ezsi-scram cells were compared in xenograft models. Spontaneous and experimental metastasis in mouse models were examined to study the role of ezrin in the growth and metastasis of MiaPaCa-2 cells *in vivo*. In the spontaneous metastasis models, the tumor incidences were 100% (8/8) in the Mia ez22-B, Mia pcb6, Mia ezsi-E and Mia ezsi-scram cell-treated animals. The body and tumor weight of the experimental animals showed no apparent differences among the four cell clone-treated animals (*P *> 0.05) (Table 1). Six out of the eight nude mice treated with the Mia ez22-B cells developed mesentery lymph node metastasis, whereas only one out of the eight Mia pcb6-treated mice developed mesentery lymph node metastasis (*P *< 0.05). In addition, one out of the eight Mia ez22-B-treated mice displayed a diaphragm metastasis. Moreover, one out of the eight Mia ezsi-scram-treated mice developed mesentery lymph node metastasis, whereas no metastasis was found in the Mia ezsi-E-treated animals (P > 0.05); none of the four groups was found to be present with internal organ metastasis (Table 1). In the experimental metastasis mouse models, two out of the eight Mia ez22-B-treated mice exhibited tumor metastasis, with one metastasis found in the spinal cord and the other in the pelvic cavity and adrenal gland area. No metastasis was found in the nude mice treated with the other three cell lines (*P *> 0.05). These data indicate that ezrin overexpression can induce metastasis *in vivo *in spontaneous metastasis mice models; however, ezrin silencing had no obvious effect on the metastatic potential of MiaPaCa-2 cells.

**Table 1:** Ezrin induces enhanced tumor metastasis *in vivo*

groups	body weight (g)		tumor weight(g)		tumor incidence	metastasis		
	average	range	average	range		MLN	Dia	IO
Mia pcb6	18.14	13.9-20.7	3.08	1.55-4.95	8/8	1/8	0/8	0/8
Mia ez22-B	20.94	15.7-24.2	3.84	1.17-5.80	8/8	6/8*	1/8	0/8
Mia ezsi-scram	18.35	13.5-21.3	3.22	2.50-5.25	8/8	1/8	0/8	0/8
Mia ezsi-E	18.84	14.0-25.0	3.02	2.30-4.23	8/8	0/8	0/8	0/8

MLN: mesentery lymphoid nodes Dia: diaphragm IO: internal organs

* Statistically different (P < 0.05)

### Immunohistochemical analysis of ezrin expression in pancreatic ductal carcinoma samples

**Figure 7:**
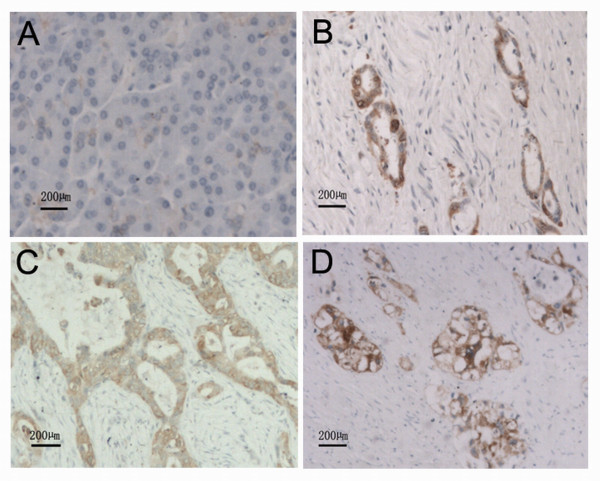
**Ezrin expression in normal pancreatic tissue and pancreatic ductal adenocarcinoma shown by immunohistochemistry.** (A) Normal pancreatic tissue. (B) Well-differentiated pancreatic ductal adenocarcinoma. (C) Moderate-differentiated pancreatic ductal adenocarcinoma. (D) Poor-differentiated pancreatic ductal adenocarcinoma.

To study the role of ezrin in pancreatic cancer, we analyzed its expression pattern in 70 PDAC patients and 61 normal pancreatic or paraneoplastic tissues (more than 1.5 cm away from the tumor). Ezrin was not detectable in normal pancreatic ducts and acini (Figure 7A); however, 64 PDAC samples were found to be ezrin positive (91.4%, 64/70) (Figure 7B-D, Table 2), suggesting that ezrin was overexpressed in human PDAC and that ezrin expression was likely associated with pancreatic cancer development. To determine whether or not ezrin expression was correlated with any clinical-pathological parameters, the relationship between ezrin expression and histological grading, as well as clinical staging was analyzed. We found that ezrin expression was not correlated with histological grading, pathologic stage, lymph node status or the depth of invasion (Table 2).

**Table 2:** Association between ezrin expression and clinico-pathologic variables in 70 patients with pancreatic ductal adenocarcinoma

variable	No. of patients	ezrin expression		P*
			
		positive(n = 64)	negative(n = 6)	
Age				0.175
< 65	53	47	6	
> 65	17	17	0	
Gender				0.34
Male	45	41	4	
Female	25	23	2	
Histopathogic grading				0.4688
G1	8	8	0	
G2	38	34	4	
G3	24	22	2	
Depth of invasion				0.653
T1	2	2	0	
T2	17	15	8	
T3	46	42	4	
T4	5	5	0	
Pathologic stage				0.249
I	5	5	0	
II	16	14	2	
III	41	39	23	
IV	8	6	2	
LN metastasis				0.3304
Negative	27	25	2	
Positive	43	39	4	

LN:Lymph node

*Fisher's exact test was used for analysis

### Ezrin expression in the tubular complexes in CP and PanIN, as well as in the proliferated intercalated ducts in the pancreatic tissue adjacent to PDAC

**Figure 8:**
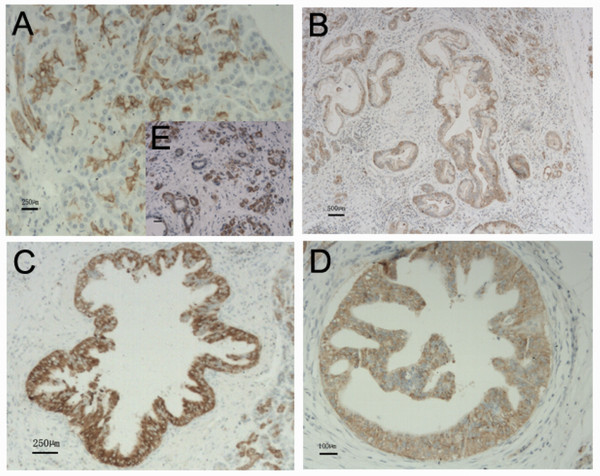
**Expression of ezrin in intercalated duct cells, the tubular complexes of CP and PanINs.** (A) Staining of ezrin in the intercalated duct cells in the paraneoplastic tissues. (B-D) Ezrin-positive staining in the representative clinical specimens of PanIN1, PanIN2 and PanIN3 (×100 magnification). (E) Ezrin expression in the cells of the tubular complexes in CP (×100 magnification).

We then investigated the role of ezrin in precancerous lesions, including the tubular complexes in CP and PanIN that are considered to be precancerous lesions of PDAC. In total, 24 out of 28 (85.7%) samples displayed positive staining of ezrin in the tubular complexes (ductal-like cells, Figure 8E). 33 out of 34 PanIN cases (97.1%) were ezrin positive (Figure 8B-D). As previously reported, PanIN can be classified into three main stages (1, 2 and 3) based on hyperplasia status and morphology of the epithelial cells. In this study, 9 PanIN-1, 13 PanIN-2 and 12 PanIN-3 samples were examined. Ezrin expression was observed in 8/9 (88.9%) of the PanIN-1 cases, 13/13 (100%) of PanIN-2 and 12/12 (100%) of PanIN-3 (Figure 8B-D). No significant differences in ezrin-positive staining were found among the three classes of PanIN lesions (*P *> 0.05). We also observed that ezrin was expressed in the intercalated duct cells (Figure 8A) in pancreatic tissue adjacent to the adenocarcinoma. These results indicate that ezrin expression is associated with early stages of pancreatic cancer development.

## Discussion

Ezrin is the best characterized member in the ERM family; it shares the common membrane-binding N-terminal FERM domain with band-4.1 family members [32]. Ezrin linking the cell membrane to actin cytoskeleton allows a cell to interact with its microenvironment and provides an "intracellular scaffolding" that facilitates signal transduction through a number of growth factor receptors and adhesion molecules [2,11,33]. Positioned at the cell membrane-cytoskeleton interface, ezrin may be a nexus in the metastatic phenotype, playing a central, necessary and early role in the process of metastasis [22]. Upon threonine and tyrosine phosphorylation, ezrin assumes an active, "open" conformation and, in turn, moves to the cell membrane and directly or indirectly tethers F-actin to the cell membrane. Ezrin resides at the nexus of multiple pathways regulating cellular behavior that can influence metastatic potential, including cell survival, motility, invasion and adherence. Ezrin participates in several crucial signal transduction pathways, including the MAPK, AKT, Rho kinase and CD44 pathways, promoting cytoskeletal reorganization and subsequent morphogenetic alterations [3,5,8,11]. High-level ezrin expression was observed in many tumor cell lines, such as breast carcinoma and rhabdomyosarcoma cell lines [19-21]. Ezrin overexpression was also been observed in borderline lesions and pancreatic cancer tissues and associated with tumor malignant transformation and metastatic potential [23-26]; however, its role and mechanisms remain elusive.

The invasion of cells into the surrounding tissue is a multi-step action that requires cell-cell contact, cell motility and degradation of the extracellular matrix by matrix metalloproteinases [34,35]. Here we demonstrated that ezrin was involved in the cytoskeleton modulation by SEM, showing the ezrin-induced changes in cell protrusions, cell microvilli and pseudopodia compared to the control cells. Consistent with these results, the chamber migration and invasion assays confirmed that ezrin expression could alter the cell migration and invasion abilities of pancreatic cancer cells. Ezrin is a cytoskeletal protein that might affect the assembly of cytoskeletal elements at the cytoplasmic face of the membrane and the nuclear skeleton, which would then facilitate cell migration and invasion. These results were in agreement with the previous reports demonstrating that changes in cytoskeleton might be a key factor in regulating neoplastic progression and tumor growth [13,22,32,36].

Our results showed that increased level of the ezrin protein was correlated with an increase in anchorage-independent growth of tumor cells, consistent with the previous finding in glioma cells [13]. We also established experimental and spontaneous mice models and showed that ezrin overexpression could enhance tumor metastasis *in vivo*, consistent with our observations in the cell motility/invasion and soft agar colony formation assays *in vitro*.

Our results also showed that ezrin overexpression could induce metastasis *in vivo *in the spontaneous metastasis mouse model; however, ezrin silencing exerted no obvious effect on the metastatic potential of MiaPaCa-2 cells. These observations might be explained by the fact that MiaPaCa-2 cells is a cell line with low metastatic potential; therefore, the effect of ezrin silencing on metastasis may not be obvious. In addition, ezrin silencing might affect other signal pathway.

A striking feature of ezrin overexpression was the increased formation of surface protrusions that play essential roles in cell motility [37]. Aside from the increased formation of protrusions, we proposed another possible mechanism for the effects of ezrin on cell invasion. Our results showed that phosphorylated Erk1/2 was markedly increased in the Mia ez22-B cells, although no significant changes were observed in the Mia ezsi-E cells compared to the controls. Activation of Erk1/2 by ezrin in MiaPaCa-2 cells indicates that the Erk1/2 MAPK pathway is one of the mediators of ezrin signaling. Therefore, the Erk1/2 pathway may also be involved in ezrin-induced cell motility, invasion and morphological changes in pancreatic ductal adenocarcinoma. This result is consistent with previous studies showing that the Erk1/2 pathway is involved in the reappearance of the actin cytoskeleton, allowing for the extension of ruffles into the active protrusions that are required for cell motility and, therefore contributing to the alteration of cell motility and invasion [29,30]. Although the phosphatidylinositol 3-kinase/Akt pathway is involved in ezrin-mediated cell survival [11], we did not find corresponding evidence in either the ezrin-overexpressing or the ezrin-silencing MiaPaCa-2 cells. Although a recent study has shown that overexpression of pEzrin(Tyr353) in pancreatic cancers is associated with positive lymph node metastasis, less differentiation, pAkt overexpression, and shorter survival times [27], we did not observe any change of ezrin phosphorylation in either the ezrin overexpressing or the ezrin silencing MiaPaCa-2 cells. Therefore, phosphorylation of ezrin may not affect the motility and invasion ability of MiaPaCa-2 cells *in vitro*. Ezrin overexpression may be sufficient to confer metastatic potential [38], and ezrin silencing may reverse metastatic behavior, through other ways [21]. These underlying mechanisms require further elucidation.

Immunohistochemical analysis demonstrated that ezrin expression was elevated in PDAC samples compared to normal pancreatic tissues, which provided additional evidence supporting a functional role of ezrin in pancreatic cancer development. We also observed that ezrin was highly expressed in precancerous lesions, such as PanINs (97.1%, 33/34) and tubular complexes in CP (85.7%, 24/28). These observations have further significance. The detection and treatment of early-stage, non-invasive PanINs has a major impact on pancreatic cancer survival. PanINs are morphologically classified into three grades, according to nuclear polarity, nuclear size (pleomorphism) and hyper-chromatic staining [39,40]. Despite complex associations between tumor cells and PanINs, histological and molecular evidence suggests that PanINs can gradually progress to PDAC, bearing genetic traits such as Ras mutations, Cyclin D1 overexpression and loss of p16 expression [41,42]. Because CP is also an independent risk factor for PDAC development [41], high ezrin expression proportion in CP and PanINs suggests that ezrin might be involved in the earliest stages of PDAC pathogenesis and could potentially serve as an indicator for those lesions progressing to the more advanced stage--PDAC. Ezrin was also expressed in the intercalated ducts in the pancreatic tissue that was adjacent to the adenocarcinoma, which was considered to be the origin in the pancreatic ducts and acini, as well as the starting point of PDAC development [43,44]. The results indicate that ezrin may play an important role in the early development of pancreatic ductal carcinoma.

In conclusion, we successfully overexpressed and silenced the ezrin protein expression in MiaPaCa-2 cells, and found that changes in the ezrin protein level were correlated with changes in the formation of dynamic cell protrusions, motility, invasion and the ability of anchorage-independent growth, which are all tumor cells features. Based on these results, we propose that ezrin, by participating in the formation of cell protrusions and the enhancement of anchorage-independent growth ability, might promote invasion and metastasis in carcinogenesis. These processes may be attributed to the activation of the Erk1/2 pathway [29,30]. The high ezrin expression proportion in CP, PanINs and ezrin expression in intercalated duct cells suggest that ezrin might be involved in the earliest stages of PDAC pathogenesis and could potentially serve as an indicator for those lesions progressing to the more advanced stage, PDAC. These results indicate that blocking ezrin function may represent a novel and effective strategy for preventing pancreatic cancer progression, invasion and metastasis. In pancreatic precancerous lesions, such as PanINs and chronic pancreatitis, blocking ezrin function may have therapeutic effects that prevent these two diseases from progressing to pancreatic cancer.

## Conclusions

We propose that ezrin might play functional roles in modulating morphology, growth, motility and invasion of pancreatic cancer cells, and that the Erk1/2 pathway may be involved in these roles. Moreover, ezrin may participate in the early events of PDAC development and may promote its progression to the advanced stage.

## Competing interests

The authors declare that they have no competing interests.

## Authors' contributions

YM participated in the design of the study, performed experiments, analyzed the data and drafted the manuscript. ZL performed the immunohistochemical evaluations and participated in writing the manuscript. SY contributed to study design and conducted the animal studies. QZ performed the experiments, analyzed the data and drafted the manuscript. YM contributed to data analysis. JC planned the study, supervised the statistical calculations, performed the immunohistochemical evaluations, coordinated the study and drafted the manuscript. All authors read and approved the final manuscript.
